# Electronic and magnetic properties of Co doped MoS_2_ monolayer

**DOI:** 10.1038/srep24153

**Published:** 2016-04-07

**Authors:** Yiren Wang, Sean Li, Jiabao Yi

**Affiliations:** 1 School of Materials Science and Engineering, UNSW, Sydney, 2052, Australia

## Abstract

First principle calculations are employed to calculate the electronic and magnetic properties of Co doped MoS_2_ by considering a variety of defects including all the possible defect complexes. The results indicate that pristine MoS_2_ is nonmagnetic. The materials with the existence of S vacancy or Mo vacancy alone are non-magnetic either. Further calculation demonstrates that Co substitution at Mo site leads to spin polarized state. Two substitutional Co_Mo_ defects tend to cluster and result in the non-magnetic behaviour. However, the existence of Mo vacancies leads to uniform distribution of Co dopants and it is energy favourable with ferromagnetic coupling, resulting in an intrinsic diluted magnetic semiconductor.

Graphene is one of the two dimensional (2D) materials, which has shown many extraordinary properties in a variety of research areas, such as energy materials, catalyst, and spintronics. The success of the research in graphene^1^ has inspired many interests in studying other single-layer two-dimensional (2D) materials like boron nitride (BN), silicene, and transition metal dichalcogenides (TMDCs)^2^,^3^,^4^,^5^,^6^,^7^. These 2D materials have also demonstrated great potential for the new generation low-dimensional transistors, photo-emitting devices and spintronics devices due to their unique structural and electronic properties.

Among these materials, TMDCs covers a wide range of materials including MoS_2_, WS_2_, TiSe_2_, TiS_2_, VS_2_, ZrS_2_, HfS_2_, NbS_2_, TaS_2_, TiSe_2_, VSe_2_, and NbSe_2_ *etc*. MoS_2_ is one of the most studied 2D materials besides graphene, which has attracted broad attention^7^,^8^,^9^,^10^. Similar to graphene, the monolayer MoS_2_ (1H-MoS_2_) exhibits unique properties from its bulk counterpart. For example, the bulk MoS_2_ is an indirect band gap semiconductor with a band gap of 1.29 eV while the monolayer has a direct band gap of 1.8 eV^11^. 1H-MoS_2_ can be fabricated via different methods like mechanical or liquid exfoliation, chemical vapour deposition and electron irradiation^12^,^13^,^14^. Different from graphene with zero bandgap and light carbon atom nature resulting in a very low spin-orbital coupling, MoS_2_ has a relative strong spin-orbital coupling, thus making the spin manipulation possible for the applications of spintronics devices^10^. Theoretically, quantum Hall effect, spin Hall Effect and spin manipulation by electric field have been predicted by first principle calculations^15^,^16^,^17^. Experimentally, high spin injection efficiency has been achieved using Fe as the electrode^18^. In particular, the carrier mobility of exfoliated TMDC is relatively low comparing to graphene, therefore can limit the applications of such kind of materials^19^,^20^. However, a record high electron mobility of fewer-layer MoS_2_ is measured to be 34,000 cm^2 ^V^−1 ^s^−1^ at low temperature using a heterostructure device platform^21^. These findings further confirm the possibility of fabricating monolayer MoS_2_ spintronics devices.

Though abundant researches have been reported on the electrical, mechanical and optical properties of monolayer MoS_2_ experimentally and theoretically^20^,^22^,^23^,^24^,^25^,^26^, the studies on the magnetic properties of MoS_2_ are still very limited^27^. In fact, the pristine bulk MoS_2_ is nonmagnetic. However, recent theoretical and experimental results indicate that MoS_2_ nanostructures are magnetic and the magnetic moment mainly comes from the zigzag edges or vacancies^28^,^29^. In addition, Togay *et al*.^28^ discovered that the magnetic measurements were insensitive to the interlayer coupling and therefore proposed that the monolayer MoS_2_ might share the same magnetic behaviours and can be possible diluted magnetic semiconductor (DMS).

Inspired by the experimental studies, theorists employed first principle calculations to investigate the magnetic behaviour of MoS_2_ monolayer. As a matter of fact, the calculation results show that similar to its bulk counterpart, the pristine MoS_2_ monolayer is also nonmagnetic^30^. In this case, the impurity absorption on the surface of MoS_2_ monolayer has been strategically used to modify the electronic and magnetic properties. First principle calculation results indicate that the absorption of B, C, and N on 1H- MoS_2_ leads to the ferromagnetic ordering while H and F absorptions induce weak antiferromagnetic coupling^31^. On the other hand, the adsorption of transition metal atoms, such as Co, Cr, Fe, Ge Mn, Mo, Sc and V, result in a local magnetic moment^32^. Besides the adsorption of impurity atoms, strain in the monolayer is also responsible for the magnetic properties. The calculations demonstrate that stain cannot induce any magnetism in the pristine monolayer MoS_2_^33^,^34^,^35^,^36^. However, the magnetic moment in MoS_2_ monolayers can be generated by the particular native defects when tensile strain is applied^36^,^37^. Therefore, the defects should be considered for the study of magnetic properties of MoS_2_ monolayers.

In the research of oxide based DMSs, doping is one of the most important techniques to realize the ferromagnetism at room temperature. Magnetic and nonmagnetic element dopants have both been successfully used to tune and tailor the semiconductor properties^38^,^39^,^40^,^41^,^42^,^43^. Similarly, from the first principle calculations, magnetic moment is produced when monolayer MoS_2_ is doped with non-metals (H, B, N and F) or transition-metals (V, Cr, Mn, Fe and Co) to substitute the S^44^. Theoretical calculations have also shown that the partial replacement of Mo with the transition-metals including Mn, Co, Fe, and Zn creates the magnetism in MoS_2_ as well^45^,^46^,^47^,^48^. However, the reported results in this area are contradictive. As mentioned before, the magnetism in MoS_2_ nanostructures can be much influenced by the zigzag edges, while experimental and computational study has shown the magnetism in Co doped MoS_2_ nanosheets is sensitive to the edges as well^49^. The samples with 0%, 3%, 5%, and 7% Co doping are found to be ferromagnetic and the magnetization weakens with the increase of doping concentrations. DFT calculations are performed with MoS_2_ nanoribbon structures and the largest magnetic moments are achieved in pure MoS_2_ model. However, this result is very different from the others results in monolayer MoS_2_. The pristine MoS_2_ monolayer is found to be nonmagnetic while Co doped MoS_2_ has been reported to be antiferromagnetism or ferromagnetism at different calculations^45^,^46^,^47^,^48^. In addition, the defects, such as S vacancies or Mo vacancies and their effects on the magnetic properties have not been systematically studied.

In this work, we carefully investigate the structural and electronic properties of Co doped monolayer MoS_2_ using first-principles calculations with considering the role of defects in the magnetic properties. We find that the Co dopants can induce robust magnetic moment in this system, which is associated with the d states of the Co atom and the surrounding Mo or S atoms. Moreover, we also calculate the system with different doping concentrations of Co and find out that the magnetism can only be produced when the dopants are rather dispersed and uniform, suggesting intrinsic ferromagnetism of a new kind of diluted magnetic semiconductor.

## Computational Details

First principles calculations are performed on the basis of the density-functional theory using Vienna ab-initio simulation package (VASP)^50^. Generalized gradient approximation (GGA)^51^ is used as the exchange-correlation functional together with projector-augment wave method. The plan-wave cutoff energy is set to be 475 eV. A 5 × 5 × 1 k-mesh based on gamma-centred scheme is applied for relaxation calculations and a gamma-centred 7 × 7 × 3 grid for static calculations. The relaxation convergence of energy is taken as 1.0 × 10^−5 ^eV and the Hellmann-Feynman force between each atom set to less than 0.02 eV/Å. A 4 × 4 MoS_2_ monolayer supercell structure containing 32 S and 16 Mo atoms is adopted in this calculation. For comparing the size effect of the supercell on the electronic and magnetic properties and simulating different doping levels, 3 × 3 and 5 × 5 MoS_2_ monolayer supercells with the same lattice constants and calculation details are also employed for the calculations, and their atomic structures are shown in Supplementary Fig. S1 and Fig. S2. Structural relaxation is done for supercells with and without defects. The positions of all the atoms in the supercell were fully relaxed during structural optimization. All the calculations are performed under same relaxation criteria.

Based on the previous studies^45^,^52^,^53^, the formation energy (E_f_) of a supercell with defect in neutral state is calculated to evaluate the binding strength using the following equation:


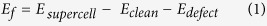


where E_supercell_ and E_clean_ stand for the total energy of the supercell with and without defect separately; E_defect_ is the energy of the isolated defect atom in the same cell size.

In order to compare the relative stabilities of defects and defects complexes before and after Co doping, the relative formation energy E_fr_ is defined by the following equation:


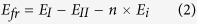


Similar to the definition of E_f_, here E_I_ and E_II_ stand for the total energy of the supercell with defect or defect complex I and II, separately; E_i_ is the energy of one isolated *i* atom which is added or removed from defect II when defect I forms; n is the number of the atoms i. A negative value of E_fr_ means the system with defect I is more stable and the defect can form easily when the defect II exists.

## Results and Discussions

### Properties of non-doped 1H-MoS_2_

**Figure 1:**
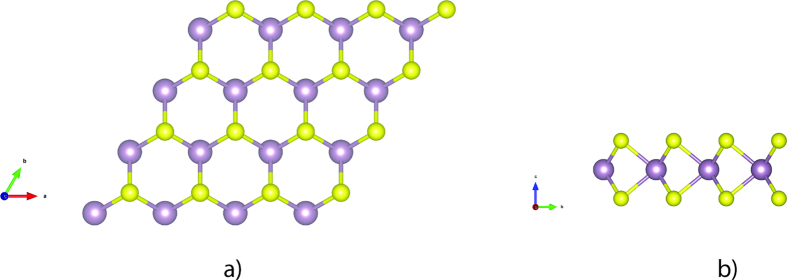
The atomic structure of 4 × 4 monolayer MoS_2_ from (**a**) top view and (**b**) side view. The big purple balls stand for the Mo atoms and small yellow balls are sulphur atoms.

**Figure 2:**
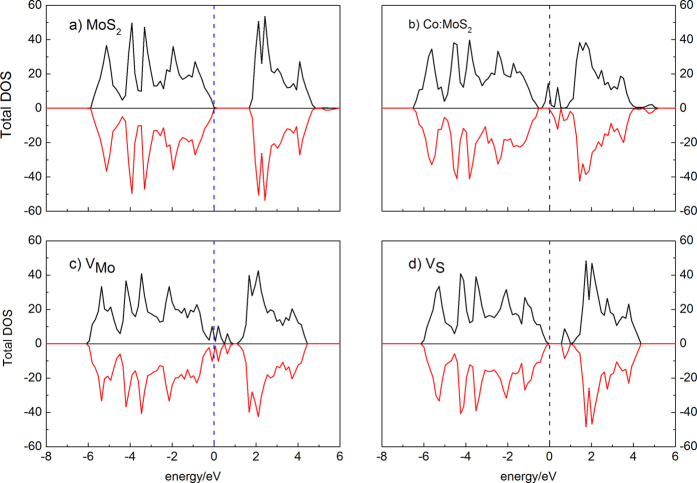
The total density of states (DOS) of (**a**) clean MoS_2_ monolayer supercell; (**b**) the supercell with one Co atom substituting Mo; (**c**) the supercell with a Mo vacancy; and (**d**) the supercell with a sulphur vacancy (the vertical dashed line indicates the Fermi level).

As shown in Fig. 1, monolayer MoS_2_ consists of three atomic layers with one Mo layer arrays between two S layers in a trigonal prismatic layout. The optimized lateral value of the supercell is 12.889 × 12.889 Å^2^. The average length of the Mo-S bond is 2.409 Å. The distance between two adjacent monolayers is set to be larger than 12 Å in order to avoid the interaction between the monolayers and their periodic images. The calculated lattice constant is 3.22 Å, agreeing well with the experimental value (3.16 Å)^54^. The calculated density of states (DOS) of the supercell demonstrated in Fig. 2(a). It shows that the calculated band gap for pristine monolayer MoS_2_ is 1.66 eV, agreeing well with the experimental value (1.8 eV) as well as others calculation result (1.65 eV^48^ and 1.67 eV^55^). The slight underestimation is due to the pseudopotential applied in this work.

### Atomic configurations of defects and defects complexes in 1H-MoS_2_

**Figure 3:**
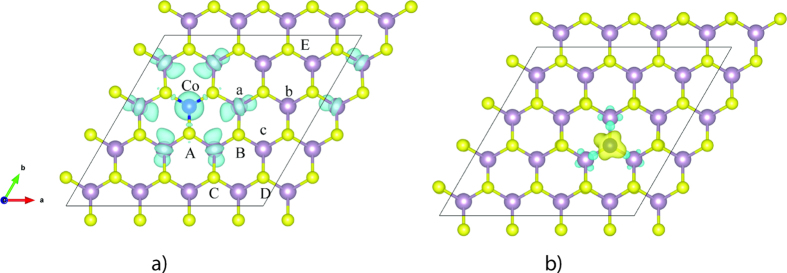
Spin densities of 4 × 4 monolayer MoS_2_ with a Co substituting Mo site (a) and S site (b). (The line denotes the 4 × 4 supercell used in calculations).

Various kinds of defects and defect complexes are considered in this study to investigate the characteristics of the doping system. First, Mo vacancy, S vacancy, or Co substitutional alone is employed to study the electronic and magnetic properties. A Mo or S vacancy is created by removing one Mo or S atom from the supercell for the calculation Subsequently, a Co substitutional is then created by filling a Co atom into a Mo or S vacancy to form substitutional Co (Co_Mo_ or Co_S_). Then, in a 4 × 4 monolayer MoS_2_ supercell, we create defects at two sites to form defect complex, namely Mo and S sites. With one defect at Mo site, there are five possible sites of the second defect at S positions, namely *A, B*, C, D and *E,* and three possible sites at the second Mo positions, namely *a, b* and *c*, as denoted in Fig. 3(a).

### Stabilities, electronic and magnetic properties of 1H-MoS_2_ with a single defect

The symmetry of the spin up and spin down bands in Fig. 2(a) confirms the nonmagnetic nature of the pristine monolayer MoS_2_. The calculations indicate that in a 4 × 4 supercell, neither a Mo vacancy nor an S vacancy is magnetic as well, since no split of the energy level near the Fermi level (which has been set to zero) can be observed in Fig. 2(c,d). The formation energy of the supercell with a single S vacancy is about 4.99 eV, which is much lower than that of a Mo vacancy (11.88 eV). Minor lattice distortion happens when the V_S_ is created: the three surrounding Mo atoms near the vacancy site will move away from their original positions of about 0.072 Å. The S atom that under the vacancy will move downwards about 0.003 Å and the six surrounding S atoms at the same atomic layer will move towards the vacancy for about 0.086 Å, while the relevant S atoms at the other layer will shift outwards for 0.029 Å. When the V_Mo_ is created, the six surrounding Mo atoms near the vacancy site will move away from their original positions of about 0.046 Å and the six surrounding S atoms from both atomic layers will move away for about 0.096 Å.

According to our calculations, Co atom can be easily embedded with the Mo vacancy since it is energetically favorable with a relative formation energy of −7.26 eV (the absolute formation energy is 4.64 eV). From the calculation results, an obvious asymmetry of the DOS of the MoS_2_ with a substitutional Co_Mo_ can be found around the Fermi level, which indicates the substitution of Co at Mo site can induce spin polarized state. The overall magnetic moment of the supercell with one Co substituting Mo is 2.99 μ_B_ and the dopant Co has a local magnetic moment of 0.90 μ_B_. The spin density for a single Co substitution at Mo site in MoS_2_ monolayer is plotted in Fig. 3(a). It is obvious to see that the Co dopant site can maintain trigonal prism symmetry after structural relaxation. The bond lengths of the Co with the surrounding S atoms and Mo atoms are 2.303 Å (Co-S bond) and 3.263 Å (Co-Mo bond). A portion of the spin densities are found to be localized at the Co atom. The spins of the six neighboring Mo atoms are coupled to the Co atom. Spin-polarized p orbitals of the S atoms can be observed as well. The six nearest-neighbor S atoms of a Co have the same spin moments. The local magnetic moments of the concerned atoms are listed in Table 1.

**Figure 4:**
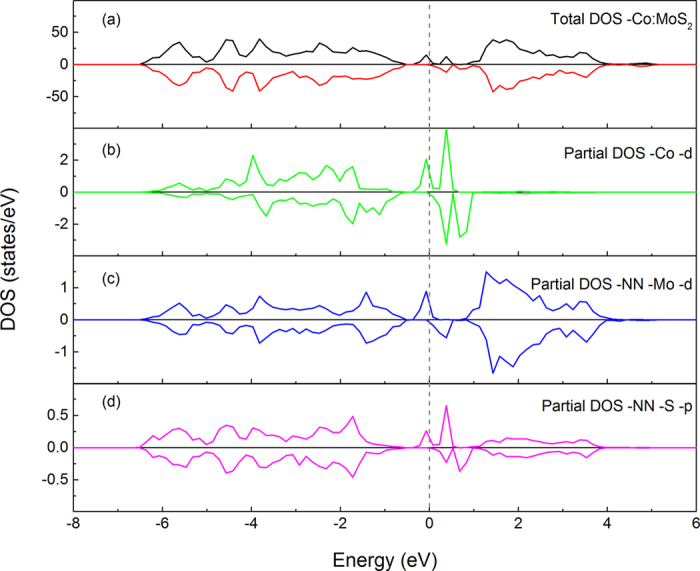
The total DOS and projected DOSs of a 4 × 4 monolayer MoS_2_ supercell with a Co atom substituting Mo site. Plane (**a**) is the total DOS, and planes (**b**–**d**) are the partial DOSs of the d orbitals of the Co atom, d orbitals of the six nearest-neighbouring Mo atoms and p states of the six nearest-neighbouring S atoms, respectively. The dashed line indicates the Fermi level.

**Figure 5:**
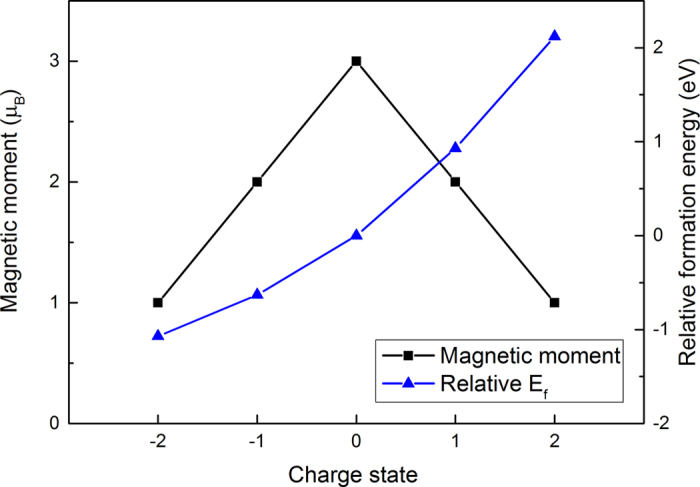
Magnetic moment and relative formation energy of Co_Mo_ in various charge states. The Fermi level of the formation energy is taken at the bottom of the valence band.

To further explore the electronic structures of the system, we calculated their projected DOSs, as shown in Fig. 4. Compared to a pristine monolayer, the monolayer doped with Co becomes half-metallic and the defect levels are formed within the MoS_2_ band gap when Co substitution occurs. The PDOSs suggest that the gap states mainly rise from the d states of the Co atom, the six spin-polarized nearest-neighboring (NN) Mo atoms and the p states of the NN S atoms in both atomic layers. Charge states have significant influence on the magnetic moment in diluted magnetic semiconductors^56^. In this work, we also investigated the effect of charge state of Co_Mo_ on the spin polarized state of the system. The results indicate that the neutral state of Co_Mo_ possesses the largest magnetic moment, as shown in Fig. 5. Both positive and negative charges lead to a decrease in magnetic moment. The formation energy is taken at the bottom of the valence band and is set relative to the formation energy of system in neutral state. From Fig. 5, it can be seen that the energy increases continuously with the Co_Mo_’s charge turning positive.

Since the formation energy of V_S_ is relatively low, the substitution of Co atom at sulfur site is taken into consideration as well. The calculation results indicate that the formation energy of Co_S_ can be lowered to 2.10 eV and it has a local magnetic moment of 1.00 μ_B_. Such magnetic moment is contributed by the d orbitals of the Co and the three surrounding Mo atoms, as depicted in Fig. 3(b). After structural relaxation, the substitutional Co atom moves about 0.04 Å upwards from the original position. The six nearby S atoms will shift about 0.05 Å close to the Co_S_ while the movement of Mo atoms is very limited from their initial sites. Though the formation energy of this defect is relatively low, it is hard to achieve anion substitution by Co doping in the experiments. In addition, the substitutional doping at Mo sites with transition metal atoms has been successfully achieved experimentally^57^. Therefore, we will focus on the Co_Mo_ related defect complexes in the subsequent calculations.

### Exploration of the size effects of the 1H-MoS_2_ supercell

**Figure 6:**
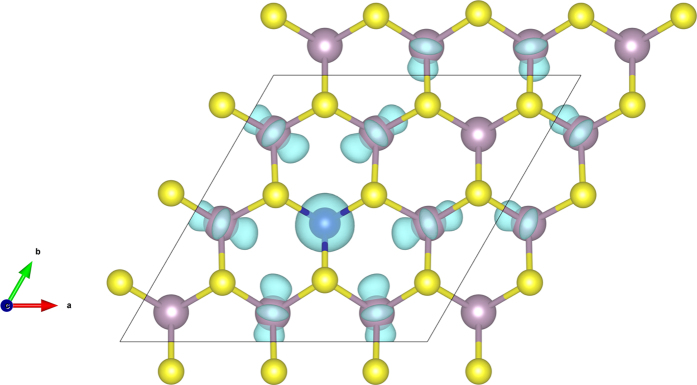
Spin density of a 3 × 3 monolayer MoS_2_ supercell with a Co atom substituting Mo site. (The line denotes the 3 × 3 supercell used in calculations).

In the 3 × 3 supercells, well convergence has reached on the formation energy of the monolayer with a single native defect of either an S vacancy or Mo vacancy. The formation energies of S vacancy and Mo vacancy are about 5.00 eV and 11.88 eV, respectively. Further calculations show that a supercell with a Mo vacancy has a total magnetic moment of 1.43 μ_B_ (spin density of this system in shown in Supplementary Fig. S3) while a sulfur vacancy does not induce any magnetic moment. Since this magnetic moment is not observed in the 4 × 4 and 5 × 5 supercells, it can be considered as the size effects in the calculations. The substitutional Co at Mo and S site can induce a magnetic moment of 2.50 μ_B_ and 0.94 μ_B_ respectively. The spin density of Co_Mo_ in 3 × 3 monolayer is plotted in Fig. 6, where most of the spins are localized in the Co atom and surrounding Mo atoms. The calculated formation energy of this defect is 4.63 eV. The dopant concentration is about 11.1 at%, suggesting that Co-doped MoS_2_ can be magnetic at high doping concentration when the Co atoms are uniform distributed in MoS_2_.

The formation energies of S vacancy and Mo vacancy in 5 × 5 supercell are 4.98 eV and 11.86 eV respectively and both vacancies do not induce magnetism. However, one Co_Mo_ can introduce a magnetic moment of 3.00 μ_B_, agreeing well with that in the 4 × 4 supercell. It is worthy to note that the doping concentration is equivalent to 4 at%. The relative formation energy of Co_Mo_ in 5 × 5 structure is about −7.22 eV compared to a single V_Mo_. With two Co atoms substituting two Mo sites, the defect complex, (Co_Mo_ + Co_Mo_), is nonmagnetic when the dopants are close to each other, which is similar to the results discussed in the 4 × 4 monolayer. Whereas, the system turns into magnetic with a magnetic moment of 6.00 μ_B_ if the two dopants are separated with a distance over 5.5 Å. All these calculations in 5 × 5 monolayers agree well with the results shown in 4 × 4 monolayers, suggesting that a 4 × 4 monolayer supercell may be suitable for the future calculations.

### Stabilities, electronic and magnetic properties of 1H-MoS_2_ with various defects complexes

Complexes with two Mo vacancies are investigated at first. The vacancy concentration is therefore increased to about 12.5 at%. The results of the formation energies, relative formation energies, and the total magnetic moments of the monolayer with different complex configurations are listed in Table 2. It is found that the complex is more stable when the two vacancies are close to each other. The formation energy of (V_Mo_ + V_Mo_) is very high indicating that this complex cannot form. In addition, the E_fr_ of (V_Mo_ + V_Mo_) relative to a single V_Mo_ is over 9.51 eV, suggesting that V_Mo_ defects prefer uniformly distributed instead of forming clustering. Moreover, this complex is nonmagnetic.

Subsequently, we calculate the complex (Co_Mo_ + V_Mo_). The results are shown in Table 3. It indicates that the two defects tend to be formed closely and this defect complex has lower relative formation energy than that to form a (V_Mo_ + V_Mo_) defect complex. This defect complex is spin polarized with a magnetic moment of 0.84 μ_B_.

We also calculate the defect complex composed of substitutional Co and S vacancy (Co_Mo_ + V_S_). The calculated results are shown in Table 4. Apparently, this defect complex has much lower formation energy when the two defects are formed closely. The total magnetic moment in this system is then 1.0 μ_B_.

Finally, we substitute two Co atoms at Mo sites in a 4 × 4 monolayer MoS_2_ supercell. The doping concentration will be increased to 12.5 at%. In this situation, the second substitutional Co_Mo_ prefers to stay in the nearest-neighbor site *a,* as shown in Table 5. Therefore, the doping atoms will be clustered via a strong thermodynamic driving force. However, we found that the complex is nonmagnetic if the two Co_Mo_ defects are clustered together. While with model *b*, the monolayer becomes magnetic with a magnetic moment of 5.63 μ_B_, this further confirms that magnetism can be achieved in Co-doped MoS_2_ with high doping concentration when the dopants are dispersed. Interestingly, when Mo vacancies exist in the system, the substitutional Co_Mo_ defects tend to distribute uniformly with a magnetic moment of 3.99 μ_B_ and it is energy favorable. This complex prefers a ferromagnetic state over antiferromagnetic state with an energy difference ΔE_FM-AFM_ = −11.3 meV. To include the strong correlation effects, we perform GGA + U calculations of the supercell with this defects complex as well.

U_Co_ = 2.5, and 3.0 eV were chosen for the calculations. The calculated relevant energy differences (ΔE_FM-AFM_) of the systems are −80.6, and −94.6 meV, respectively.

Based on the Mean-field approximation (MFA), the Curie temperature (T_C_) can be estimated from the energy difference between the system in ferromagnetic state and in antiferromagnetic state using the following equation:


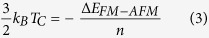


here k_B_ is the Boltzmann constant, ΔE_FM-AFM_ is the energy difference between the system in ferromagnetic state and antiferromagnetic state, and n is the number of the dopants in the supercell. The estimated Curie temperatures of U = 2.5 and 3.0 eV are 311.8 and 366 K, well above room temperature (Supplementary Information Table S1). Moreover, the systems with different U values of 2.5 and 3.0 eV are found to have same total magnetic moments of about 6.0 μ_B_. Furthermore,

A LD(S)A method on the optimized lattice structure with defects complex (Co_Mo_ + Co_Mo_) is adopted as well. The results are pretty much similar to the GGA calculations, and the system prefers a ferromagnetic state as can be seen from Supplementary Information Table S1.

The results demonstrate that the intrinsic diluted magnetic semiconductors can be achieved by doping Co in a MoS_2_ system, which is important for the potential applications in spintronics devices.

## Conclusions

From the calculations, we find that the pristine MoS_2_ monolayer does not show spin polarized state. In addition, Mo or S vacancy alone does not show spin polarized state either. However, Co substitution in Mo site produces magnetic moment. The magnetic moment originates from the d orbitals of the Co, NN Mo atoms and the p orbitals of the NN S atoms via p-d hybridization. The magnetic moment is strongly dependent on the doping concentration. Lower doping concentration (4 at% or 6.25 at%) has a stable magnetic moment of 3 μ_B_, which is higher than that at higher doping level (8 at%, or 11.1 at%, or 12.5 at%). Co_Mo_ defects tend to cluster with higher doping concentration. Subsequently the substitutional Co_Mo_ defects will not result in magnetic state. However, if Mo vacancies exist in the system, the dopants tend to separate from each other with a particular distance and the system shows ferromagnetic state. In addition, this tendency is energy favorable. The work may pave ways for achieving intrinsic diluted magnetic semiconductors based on MoS_2_ semiconductors.

## Additional Information

**How to cite this article**: Wang, Y. *et al*. Electronic and magnetic properties of Co doped MoS_2_ monolayer. *Sci. Rep.* **6**, 24153; doi: 10.1038/srep24153 (2016).

## Supplementary Material

Supplementary Information

## Figures and Tables

**Table 1:** Local magnetic moments of the dopant Co, the six neighbouring Mo and six nearest-neighbour S atoms in Co-doped 4 × 4 MoS_2_ monolayer.

	Co	Mo	S
Magnetic moments (μ_B_)	0.90	0.24	0.03

**Table 2:** Formation energies and relative binding energies of (V_Mo_ + V_Mo_) with three different configurations, based on Eqs 1 and 2.

Model (V_Mo_ + V_Mo_)	*a*	*b*	*c*
E_f_	21.39	23.38	23.48
E_b_-V_Mo_	9.51	11.51	11.60
μ_total_ (μ_B_)	0.00	0.00	0.00

The unit for the energies is eV.

**Table 3:** Formation energies and relative binding energies of (Co_Mo_ + V_Mo_) with three different configurations, based on Eqs 1 and 2.

Model (Co_Mo_ + V_Mo_)	*a*	*b*	*c*
E_f_	14.11	15.67	15.88
E_b1_-V_Mo_	9.47	11.0	11.4
E_b2_-Co_Mo_	2.23	3.80	4.00
E_b3_-(V_Mo_,V_Mo_)	−4.45	−4.88	−4.77
μ_total_ (μ_B_)	0.84	0.00	0.95

The unit for the energies is eV.

**Table 4:** Formation energies and relative binding energies of (Co_Mo_ + V_S_) with three different configurations, based on Eqs 1 and 2.

Model (Co_Mo_ + V_S_)	*A*	*B*	*C*	*D*	*E*
E_f_	7.63	9.55	18.22	9.55	9.628
E_b1_-V_S_	2.64	4.58	13.23	4.57	4.64
E_b2_-Co_Mo_	2.99	4.91	13.57	4.91	4.99
μ_total_ (μ_B_)	1.00	3.00	0.85	2.97	2.93

The unit for the energies is eV.

**Table 5:** Formation energies and relative formation energies of (Co_Mo_ + Co_Mo_) with three different configurations, based on Eqs 1 and 2.

Model(Co_Mo_ + Co_Mo_)	a	b	c
E_f_	7.65	9.16	9.05
E_b1_-V_Mo_	−4.22	−2.72	−2.83
E_b2_-Co_Mo_	3.01	4.52	4.41
E_b3_- (V_Mo_ + V_Mo_)	−13.74	−14.23	−14.43
E_b4_-V_Mo_ + Co_Mo_	−6.46	−6.52	−6.83
μ_total_ (μ_B_)	0.00	5.63	3.99

The unit for the energies is eV.
