# Supplementary material for: Electronic and magnetic properties of Co doped MoS2 monolayer
Source: Sci Rep. 2016 Apr 7;6:24153. doi: 10.1038/srep24153 (PMC4823719; doi:10.1038/srep24153)
Supplement: Supplementary Information [file srep24153-s1.pdf]

(Supplementary Information)

Electronic and magnetic properties of Co doped MoS_2 monolayer

Yiren Wang[1], Sean Li[1], Jiabao Yi[1,*]

[1]School of Materials Science and Engineering, UNSW, Sydney, 2052, Australia

[*]Tel: 61-293854837; Fax: 61-293856565; Email: Jiabao.yi@unsw.edu.au

1. The atomic structures of 3×3 and 5×5 1-H MoS_2 supercells

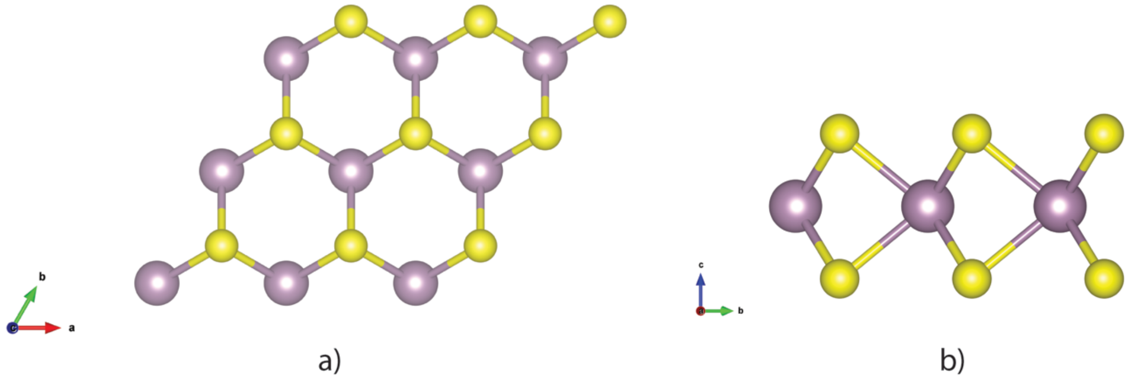

Figure S1 The atomic structure of 3×3 monolayer MoS_2 from (a) top view and (b) side view.

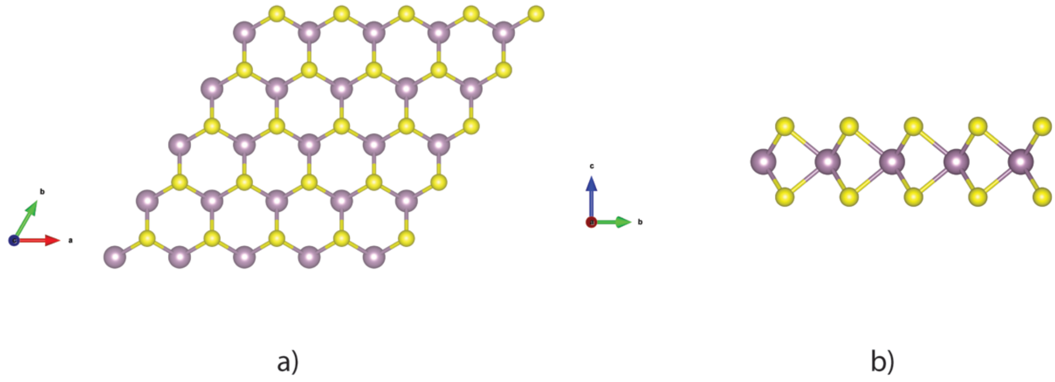

Figure S2 The atomic structure of 5×5 monolayer MoS_2 from (a) top view and (b) side **view.**

2. Spin density of 3×3 1H-MoS_2 with a Mo vacancy

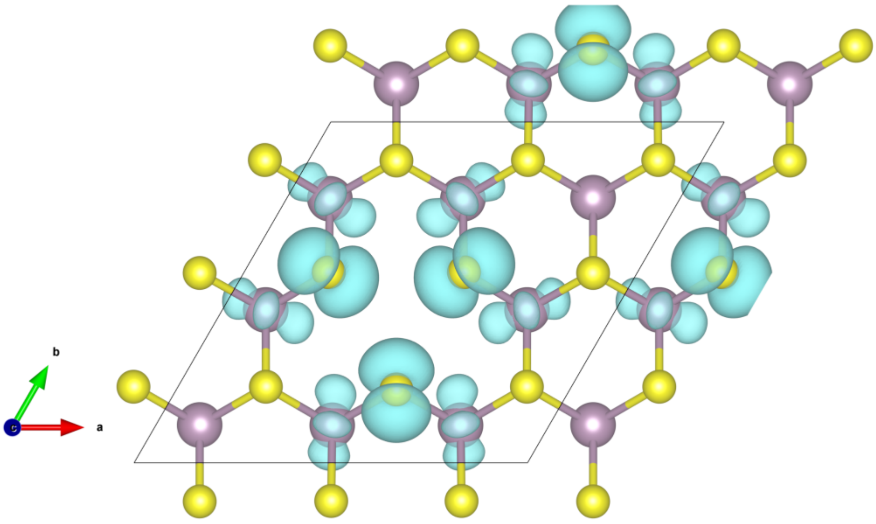

Figure S3 The spin densities of 3×3 monolayer MoS_2 with a Mo vacancy. The line denotes the 3×3 supercell.

3. The calculated Curie temperature of 4×4 1-H MoS_2 supercells using different methods

Based on the Mean-field approximation (MFA), the Curie temperature (T_C) can be estimated from the energy difference between the system in ferromagnetic state and in antiferromagnetic state using the following equation:

$$\frac{3}{2}k_B T_C = -\frac{\Delta E_{FM-AFM}}{n} \quad (1)$$

Here k_B is the Boltzmann constant, $\Delta E_{FM\text{-}AFM}$ is the energy difference, and n is the number of the dopants in the supercell which corresponds to 2 in this case.

To include the strong correlation effects GGA+U calculations of the supercell with this defects complex (Co_{Mo}+ Co_{Mo}) are performed. A fixed U=2.50 eV is adopted for Mo atoms based on the previous studies, series values of U of Co are chosen from 0 to 3.0 eV. Based on the Eq.1 from MFA, the Curie temperature can be obtained as shown in the Table S1.

LD(S)A method on optimized lattice structure with defects complex (Co_{Mo}+ Co_{Mo}) is adopted as well. The results are pretty much similar with the GGA calculations, and the system prefers a ferromagnetic state as can be seen from Table S1.

Table S1 Energy difference ΔE (in meV) between ferromagnetic and antiferromagnetic ordering (EFM − EAFM) of t defects complex (CoMo+ CoMo) in position c with GGA+ U and LDA methods. Difference values of Hubbard U (in eV) for the 3d electrons in Co are adopted in GGA+ U method. A negative energy corresponds to FM ordering being more stable.

Method	GGA+ U_{Co}=0	GGA+ U_{Co}=2.5	GGA+ U_{Co}=3.0	LDA
ΔE(eV)	-11.3	-80.6	-94.6	-11.4
T_C (K)	43.7	311.8	366	43.8
